# Enhanced Rb/E2F and TSC/mTOR Pathways Induce Synergistic Inhibition in PDGF-Induced Proliferation in Vascular Smooth Muscle Cells

**DOI:** 10.1371/journal.pone.0170036

**Published:** 2017-01-11

**Authors:** Yue Li, Xuan Li, Jie Liu, Wei Guo, Hongchao Zhang, Jianchang Wang

**Affiliations:** 1 Department of Cardiac surgery, Air Force General Hospital of People`s Liberation Army, Beijing, China; 2 Department of Vascular and Thyroid Surgery, 1^st^ Hospital of China Medical University, Shenyang, Liaoning, China; 3 Department of Vascular and Endovascular Surgery, General Hospital of People`s Liberation Army, Beijing, China; 4 Vascular Surgery Research Laboratories, Division of Vascular and Endovascular Suregery, Brigham and Woman’s Hospital, Harvard Medical School, Boston, Massachusetts, United States of America; Duke University, UNITED STATES

## Abstract

Platelet-derived growth factor (PDGF) plays an essential role in proliferation of vascular smooth muscle cells (VSMCs). The Rb/E2F and TSC/mTOR pathways contribute to the proliferation of VSMCs, but its exact roles in PDGF-induced proliferation are unclear. In this study, we demonstrated the roles of Rb/E2F and TSC/mTOR pathways in PDGF-induced proliferation in VSMCs. We found that PDGF stimulates the activity of E2F and mTOR pathways, and knockdown of either Rb or TSC2 increases PDGF-induced proliferation in VSMCs. More interestingly, we revealed that enhancing both E2F and mTOR activity leads to synergistic inhibition of PDGF-induced proliferation in VSMCs. We further identified that the synergistic inhibition effect is caused by the induced oxidative stress. Summarily, these data suggest the important regulations of Rb/E2F and TSC/mTOR pathways in PDGF-induced proliferation in VSMCs, and also present a promising way to limit deregulated proliferation by PDGF induction in VSMCs.

## Introduction

Phenotypic switching of vascular smooth muscle cells (VSMCs) is a critical step in the regulation of vascular function in health and disease. In normal condition, VSMCs exhibit quiescent status, and proliferate at a very low rate. However, upon vascular injury, VSMCs undergo a transition to pathophysiologic synthetic status, and proliferate at a high rate. Proliferation of VSMCs contributes to the pathogenesis of intimal hyperplasia and atherosclerosis. In response to vascular injury, VSMCs release various growth factors and cytokines including platelet-derived growth factor (PDGF). Activation of the PDGF pathway is known to promote status modulation of VSMCs which lead to increased cell proliferation and migration [1,2,3,4].

Cell proliferation shares a final common pathway: cell cycle. Rb/E2F pathway plays a central role in regulating cell cycle. Previous studies suggest that Rb/E2F pathway is involved in the proliferation of VSMCs. Overexpression of p21 inhibited the phosphorylation of Rb and reduced neointimal hyperplasia [5]. Transduction of porcine femoral arteries with an adenoviral vector expressing a nonphosphorylatable, constitutively active form of Rb significantly reduced neointima formation, presumably through the inhibition of E2F activity [6]. In addition to Rb/E2F pathway, TSC/mTOR pathway also contributes to the regulation of cell cycle protein expression by controlling protein translation and synthesis. Studies showed that rapamycin, which blocks the activity of the mTOR, inhibited the proliferation of VSMCs in vitro and in vivo. Rapamycin administration in pig significantly reduced the arterial proliferative response after percutaneous transluminal coronary angioplasty (PTCA) by increasing the level of cyclin-dependent kinase inhibitor p27^kip1^ and inhibition of the Rb phosphorylation within the vessel wall [7]. Clinical trial with rapamycin-coating stents showed no restenosis occurred in patients receiving rapamycin-coating stents in 1 year as compared with 26% in patients receiving placebo [8]. These evidence suggest that Rb/E2F and TSC/mTOR pathways may work together to play important roles in PDGF-induced proliferation in VSMCs. However, the molecular mechanisms are still unclear.

In this study, we characterized the mechanisms of Rb/E2F and TSC/mTOR pathways in PDGF-induced proliferation in VSMCs. We first found that both Rb/E2F and TSC/mTOR pathways are induced by PDGF treatment. We then manipulated the pathway in human primary VSMCs and Rat A7r5 cells by knockdown of Rb or TSC2 using shRNA or CRISPR system, and studied the effects in PDGF-induced proliferation. We found that knockdown of either Rb or TSC2 increases PDGF-induced cell proliferation. However, knockdown of both Rb and TSC2 caused synergistic inhibition in PDGF-induced proliferation. And we further identified that the synergistic inhibition is due to enhanced reactive oxygen species (ROS) in cells. And the gene expressions of ROS scavenger enzymes and components in cell survival/proliferation signaling were significantly downregulated in cells with double knockdown of Rb and TSC2. Together, we demonstrated the regulations of E2F/Rb and TSC/mTOR pathways in PDGF-induced proliferation in VSMCs, and the synergistic inhibition effect we found provides insight to limit unregulated VSMCs proliferation.

## Materials and Methods

### Cell Culture

A7r5 cells were obtained from the American Type Culture Collection (Rockville, MD), and cultured in Dulbecco's modified Eagle's medium supplemented with 10% fetal bovine serum (FBS), 50 IU penicillin/streptomycin, and 2 mmol/l L-glutamine from Invitrogen (Carlsbad, CA). Human aortic smooth muscle cells were purchased from Lonza (Basel, Switzerland), and cultured in smooth muscle basal medium (Lonza) supplemented with 1% human epidermal growth factor, 1% insulin, 0.2% human fibroblast growth factor B, and 5% FBS. All the cells were maintained in a humidified atmosphere with 5% CO2 at 37°C. Recombinant human PDGF-BB was purchased from PeproTech (Rocky Hill, NJ). Cells were stimulated with 10 ng/ml of PDGF-BB for 48 hours [9].

### Plasmids and Lentiviral Preparation and Transduction

The pLKO.1 lentiviral RNAi expression system was used to construct lentiviral shRNA. The sequences of shRNA used in this study is described in previous study [10]. The lentiCRISPRv2 expression system was used to construct lentiviral CRISPR for Rb and TSC2. The sequences of Rb CRISPR: Rb Oligo1: 5’-CACCGGAGAGAGAGCTTGGCTAACG-3’, Rb Oligo2: 5’-AAACCGTTAGCCAAGCTCTCTCTCC-3’. The sequences of TSC2 CRISPR: TSC2 Oligo1: 5’-CACCGGCGGCCTCAACAATCGAATC-3’, TSC2 Oligo2: 5’-AAACGATTCGATTGTTGAGGCCGCC-3’ [11]. Production of lentivirus was performed as described [10]. Single clone was established after puromycin selection. The genomic DNA of each clone was extracted for PCR to detect the indel mutation in the targeted region. The PCR products were verified by sequencing.

### RNA Isolation and qRT-PCR

Total RNA was isolated from cells using TRIzol from Invitrogen (Carlsbad, CA) for RT-PCR. Total RNA (2 μg) was reverse-transcribed using M-MLV reverse transcriptase from Promega (Madison, WI) and random primers following manufacturer’s protocol. PCR was performed in triplicate using SYBR green mix from Biotool (Houston,TX), and Real-Time PCR System from Bio-rad (Hercules, CA) under the following conditions: 3 min at 95°C followed by 45 cycles of 95°C for 20 s 60°C for 30 s and 65°C for 1 min. Primers used: human CCNA2 forward: 5’-AGTATGAGAGCTATCCTCGTGG-3’, CCNA2 reverse: 5’-CTGTAATGTACACAAACTCTGCTAC-3’; human CDK1 forward: 5’-TACAGGTCAAGTGGTAGCCATG-3’, CDK1 reverse: 5’-ACTGACCAGGAGGGATAGAATCC-3’; human CCNE1 forward: 5’-GCCAAAATCGACAGGACG-3’, CCNE1 reverse: 5’-CCCGGTCATCATCTTCTTTG-3’; human PCNA forward: 5’-GGCCAGAGCTCTTCCCTTAC-3’, PCNA reverse: 5’-TCTAGCTGGTTTCGGCTTCA-3’; human GAPDH forward: 5’-CTCTGACTTCAACAGCGACAC-3’, GAPDH reverse: 5’- CATACCAGGAAATGAGCTTGACAA -3’. Rat SOD2 forward: 5’-TTTTCTGGACAAACCTGAGCCC-3’, SOD2 reverse: 5’-AGACACAGCTGTCAGTTTCTCC-3’; Rat Catalase forward: 5’-AGAGCGGATTCCTGAGAGAGTGG-3’, Catalase reverse: 5’-TAGGAGTCCTCTTCCCAATATGC-3’; Rat Bcl2l1 forward: 5’-ACCTGAATGACCACCTAGAGCC-3’, Bcl2l1 reverse: 5’-TCATGCCCGTCAGGAACCAGCGG-3’; Rat Igf1r forward: 5’-TGGCGTCTTCACCACTCATTCC-3’, Igf1r reverse: 5’-TTGTCCAGAAGGCCGCCCTCC-3’; Rat GAPDH forward: 5’-GACATGCCGCCTGGAGAAAC-3’, GAPDH reverse: 5’-AGCCCAGGATGCCCTTTAGT-3’. The expression fold changes were analyzed by double delta Ct analysis.

### Western Blot

Cell lysate was prepared in RIPA buffer (50 mM Tris-HCl pH8.0, 150 mM NaCl, 0.1% SDS, and 0.5% Na deoxycholate, 1% NP40) with fresh proteinase inhibitor. Equal amount of protein was loaded. Western detection was carried out using a Li-Cor Odyssey image reader by software Image Studio (Ver. 2.1) (Lincoln, NE). Antibodies used: phospho-S6K (Thr389) (dilution 1:1000) from Millipore (Billerica, MA), S6K (H-9,dilution 1:2000), TSC2 (C-20, dilution 1:1000), β-Actin (AC-15, dilution 1:3000) from Santa Cruz (Dallas, TX), Rb (4.1, dilution 1:10) from Hybridoma Bank (Iowa City, IA), Goat anti-mouse/Rabbit IRDye (dilution 1:10000) from Li-Cor (Lincoln, NE).

### Transcriptional Reporter Assay

Cells were treated with lentivirus as described above, and were plated into a 24-well plate, followed by transfection by lipofetamine 2000 from Invitrogen (Carlsbad, CA) according to the manufacturer’s instruction. Luciferase activity was measure using Dual Luciferase Reporter Assay System from Promega (Madison, WI) according to the manufacturer’s instruction. Luciferase activity was read on a BD Monolight 3010 Luminometer. All data points presented are the average measurement of three independent repeats.

### Cell Proliferation Analysis

Cell proliferation was assessed by MTT assay and cell counting. For MTT assay, cells (5 × 10^3^ cells/well) were seeded into 96-well plates. After treatments described in result section, culture medium was replaced with fresh medium containing 0.5 mg/ml MTT and incubated for 2 h at 37°C. After removing the medium, 100 μl of DMSO was added to each well to solubilize the formazan present in viable cells. The plates were analyzed by measuring the optical density at 540 nm. Experiments were repeated three times. For cell counting, cells (3 × 10^5^ cells/well) were seeded into 6-well plates, and treated with indicated treatments. After grown for 5 days, living cells were counted using a hemocytometer after trypan blue staining.

### BrdU Labeling

Cells were plated on coverslips in 6-well plates. After stimulated by PDGF for 48 hours, the cells were incubated in medium containing 3 mg/ml BrdU for 2 hours at 37°C. The cells were fixed for 30 min in 4% paraformaldehyde, and permeabilized with 1×PBS with 0.1% Tween-20. After denaturing DNA for 30 min in 2 N HCl, the cells were incubated overnight with mouse anti-BrdU antibody (dilution 1:20) from Hybridoma Bank (Iowa City, IA), and then washed and incubated with FITC-anti-mouse secondary antibody (dilution 1:200) from Jackson ImmunoResearch (West Grove, PA) at room temperature for 1 hour. The coverslips were incubated with 0.3 ug/ml DAPI for 15 mins. After washed, the coverslips were mounted on glass slides with mounting medium.

### ROS Assay

ROS production in cells was measured with Dihydroergotamine (DHE) staining on coverslips. Briefly, cells were plated on coverslips in 6-well plates. After stimulated by PDGF for 48 hours, cells were incubated with medium containing 10 μM of DHE from Sigma (St. Louis, MO) for 30 minutes. The cells were fixed for 30 min in 4% paraformaldehyde, and imaged with a Zeiss fluorescence microscope. ROS production in cells was also monitored by the permeable fluorescent dyes, 5-(and-6)-chloromethyl-2'7'-dichlorodihydrofluorescein diacetateacetyl ester (H2DCFDA) from Sigma (St. Louis, MO). After the indicated treatment, cells were incubated with medium containing 10 μM of H2DCFDA for 30 minutes and then harvested and resuspended in PBS. The fluorescence intensity of intracellular DCF (excitation 488 nm, emission 530nm) was measured using FACScan from BD Biosciences (San Jose, CA).

### Statistical Analysis

The data were collected from at least three independent experiments. Values are presented as mean ± S.D. Statistical significance was assessed by Student’s two-tailed *t*-test and P<0.05 was considered statistically significant.

## Results

### Rb/E2F Pathway Is Involved in PDGF-Induced Proliferation in VSMCs

**Fig 1:**
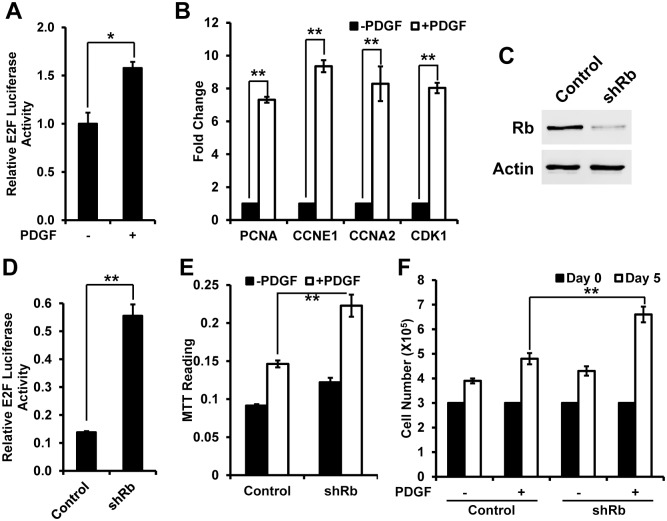
Rb/E2F pathway regulates PDGF-induced proliferation in VSMCs. (A) Measurement of E2F luciferase activity with or without PDGF stimulation. Human VSMCs were transfected with E2F luciferase constructs, and incubated with or without PDGF for 48 hrs. The E2F luciferase activity was measured by dual luciferase reporter system with BD monolight 3010 luminometer; (B) mRNA expression of Proliferating cell nuclear antigen (PCNA), Cyclin E1 (CCNE1), Cyclin A2 (CCNA2) and Cyclin-Dependent Kinase 1 (CDK1) relative to the level of GAPDH in human VSMCs with or without PDGF stimulation for 48 hrs; (C) Western blot of human VSMCs showing decreased Rb proteins after transduction with shRNA against Rb in comparison to vector control cells. Actin serves as a loading control; (D) Luciferase measurement showing increased E2F activity after transduction with shRNA against Rb in comparison to vector control cells; (E) Cell proliferation measured by MTT assay showing increased cell growth in shRb cells in comparison to vector control cells. (F) Cell proliferation measured by cell counting showing increased cell growth in shRb cells in comparison to vector control cells. *<0.05, **<0.01.

Rb/E2F pathway controls cell cycle, and plays an important role in cell proliferation. So PDGF-induced proliferation in VSMCs may be related to enhanced E2F activity. To test this hypothesis, we first compared the E2F activity in PDGF treated cells and control cells by E2F luciferase reporter assay. The results showed that PDGF significantly induced E2F luciferase activity (Fig 1A). The induced E2F activity should increase the expression of its target genes, so we next compared the transcriptional level of four well-known E2F target genes Proliferating cell nuclear antigen (PCNA), Cyclin E1 (CCNE1), Cyclin A1 (CCNA1) and Cyclin-Dependent Kinase 1 (CDK1) by real time PCR. The PCR results showed that PDGF-treated cells have significantly enhanced expression level of PCNA, CCNE1, CCNA1 and CDK1, which is consistent to enhanced E2F activity showed in luciferase assay (Fig 1B). Summarily, these results suggest that PDGF induces E2F activity.

We next want to test whether manipulating Rb/E2F pathway affects PDGF-induced proliferation in VSMCs. We used shRNA to decrease endogenous Rb level (Fig 1C). The E2F luciferase assay showed cells with knockdown of Rb have significantly higher E2F activity than control cells (Fig 1D). We next compared the proliferation between the cells under PDGF treatment. Both MTT assay and cell counting showed that cells with knockdown of Rb have a significantly higher proliferation rate than control cells (Fig 1E and 1F). Therefore, Rb/E2F pathway regulates VSMCs proliferation induced by PDGF.

### TSC/mTOR Pathway Plays an Important Role in PDGF-Induced Proliferation in VSMCs

**Fig 2:**
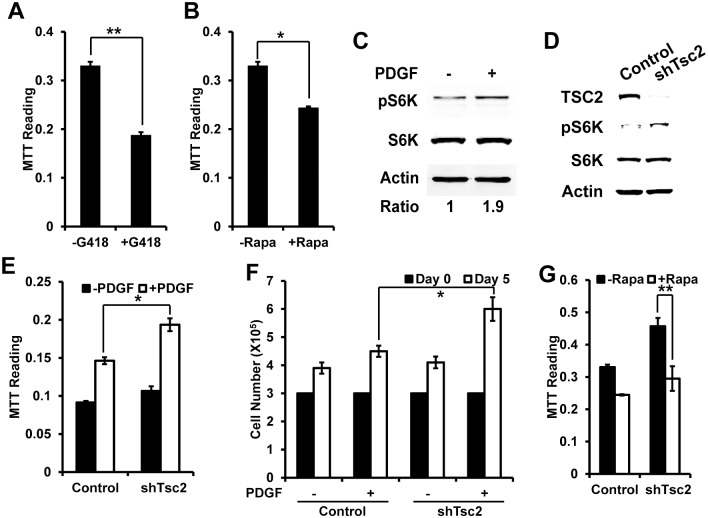
TSC/mTOR pathway regulates PDGF-induced proliferation in VSMCs. (A) and (B) Cell proliferation measured by MTT assay showing decreased cell growth in cells treated by G418 (400ug/ml) or rapamycin (10 nM) in comparison to control cells; (C) Western blot of human VSMCs showing increased phospho-S6K proteins after stimulated with PDGF in comparison to vector control cells. S6K and Actin serves as loading controls; (D) Western blot of human VSMCs showing decreased TSC2 proteins and increased phospho-S6K proteins after transduction with shRNA against TSC2 in comparison to vector control cells. (E) Cell proliferation measured by MTT assay showing increased cell growth in shTSC2 cells in comparison to control cells; (F) Cell proliferation measured by cell counting showing increased cell growth in shTSC2 cells in comparison to control cells; (G) Cell proliferation measured by MTT assay showing decreased PDGF-induced cell growth after treatment of rapamycin. *<0.05, **<0.01.

TSC/mTOR pathway involves in the regulation of protein synthesis, which is important for cell proliferation. So we hypothesized that TSC/mTOR pathway may also play a role in the PDGF-induced proliferation in VSMCs. To study this, we first treated PDGF-induced cells with or without protein synthesis inhibitor G418, and compared the cell proliferation by MTT assay. The results clearly showed that G418 significantly inhibited the proliferation induced by PDGF (Fig 2A). To connect this observation to TSC/mTOR pathway, we added mTOR inhibitor rapamycin to PDGF treated cells, and compared the cell proliferation. The data showed that rapamycin inhibited the proliferation induced by PDGF (Fig 2B). To further confirm whether treatment of PDGF increases mTOR activity, we compared the protein level of phosphor-S6K (Thr389) in cells with or without the treatment of PDGF. Phosphor-S6K is one of major downstream targets of mTOR pathway. The blot showed that the treatment of PDGF increased the phosphor-S6K level to two folds as compared to control cells (Fig 2C). In summary, PDGF induces mTOR activity.

To testing whether TSC/mTOR pathway regulates VSMCs proliferation induced by PDGF, we manipulated TSC/mTOR pathway by knockdown of TSC2 using shRNA. The western blot clearly showed the decreased TSC2 level and increased phosphor-S6K level in cells with knockdown of TSC2 (Fig 2D). Under PDGF treatment, we found cells with knockdown of TSC2 have significantly higher growth rate than control cells (Fig 2E and 2F). However, the increased growth rate is inhibited by treatment of rapamycin (Fig 2G). The results suggest that TSC/mTOR pathway plays an important role in PDGF-induced proliferation in VSMCs.

### Enhanced E2F and mTOR Activities Synergistically Inhibit PDGF-Induced Proliferation in VSMCs

**Fig 3:**
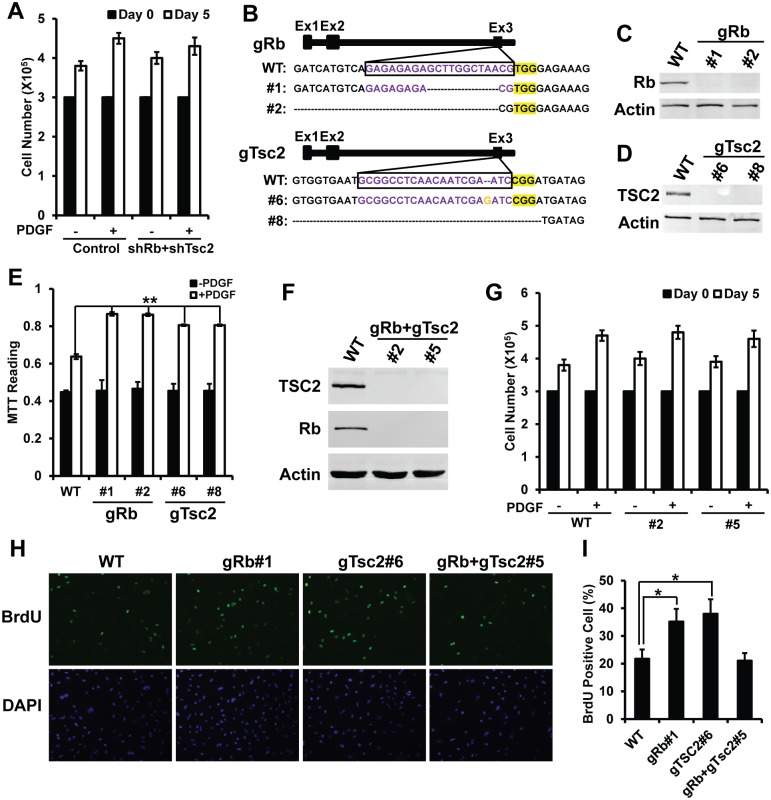
Enhanced Rb/E2F and TSC/mTOR pathways induce synergistic growth inhibition in PDGF-induced proliferation in VSMCs. (A) Cell proliferation measured by cell counting showing cell growth is back to normal after transduction with shRNA against Rb and TSC2 in comparison to vector control cells; (B) Sequence analysis of indel mutations in targeted regions of Rb (Up panel) and TSC2 (Bottom panel) locus in A7r5 cell clones. sgRNA sequences is shown in purple and the PAM sequence is in yellow. Inserted sequence is in orange. Deleted sequence is shown in dashed line; (C) and (D) Western blot of CRISPR single clones of rat A7r5 cells showing the lost expression of Rb (C) and TSC2 (D) proteins in comparison to vector control cells; (E) Cell proliferation measured by MTT assay showing increased cell growth in cells with single Rb or TSC2 knockout in comparison to control cells; (F) Western blot of CRISPR Rb and TSC2 double knockout clones showing the lost expression of both pRb and TSC2 proteins in comparison to control cells; (G) Cell proliferation measured by cell counting showing synergistic growth inhibition in cells with Rb and TSC2 double knockout in comparison to control cells; (H) Immnostaining of BrdU assay in cells with Rb and TSC2 single or double knockout under stimulation of PDGF. (I) Quantification of the percentage of BrdU positive cells in (H). *<0.05, **<0.01.

The above data revealed both Rb/E2F and TSC/mTOR pathways regulate PDGF-induced proliferation in VSMCs, and enhancing either E2F or mTOR activity increases proliferation of VSMCs. Previous studies reported that increasing both E2F and mTOR activity causes synergistic cell death in cancer cells [10]. So we are interested in testing whether similar synergistic effect is in VSMCs. To study it, we used shRNAs to knockdown the levels of both Rb and TSC2. We did not observe significantly cell death in double knockdown cells. However, in comparison to cells with single knockdown, we found that the cells with double knockdown showed decreased growth rate as control cells (Fig 3A). It suggests that enhancing both E2F and mTOR activities in VSMCs induced synergistic inhibition in cell proliferation.

To further confirm our observation in human VSMC, we applied another well-used cell model rat A7r5 cells. In A7r5 cells, we used CRISPR/Cas9 system to generate single Rb or TSC2 stable knockout cells and double stable knockout cells [12]. The sequencing data and western blots of CRISPR single cell clones confirmed the correct and complete knockout of single target genes (Fig 3B, 3C and 3D). With single knockout of Rb and TSC2 clones, we confirmed that single knockout of Rb or TSC2 increased cell proliferation under treatment of PDGF (Fig 3E). The western blots of CRISPR double knockout cell clones confirmed the lost expression of both Rb and TSC2 (Fig 3F). Cells with double knockout of Rb and TSC2 have unchanged cell proliferation as control cells (Fig 3G). The findings are consistent to our observation in human VSMCs by shRNAs. In summary, enhanced both E2F and mTOR activity causes synergistic inhibition in PDGF-induced proliferation in VSMCs.

Changes in cell proliferation might be caused by the deregulation of cell cycle and DNA synthesis. So we studied DNA synthesis in cells with Rb and TSC2 knockout by BrdU assay. The data show that cells with single Rb or TSC2 knockout have significantly more BrdU positive cells than control cells. However, cells with double knockout cells have similar percentage of BrdU positive cells as control cells (Fig 3H and 3I). It is consistent to the proliferation rate that we observed earlier.

### Enhanced E2F and mTOR Activities Induce Oxidative Stress in VSMCs

**Fig 4:**
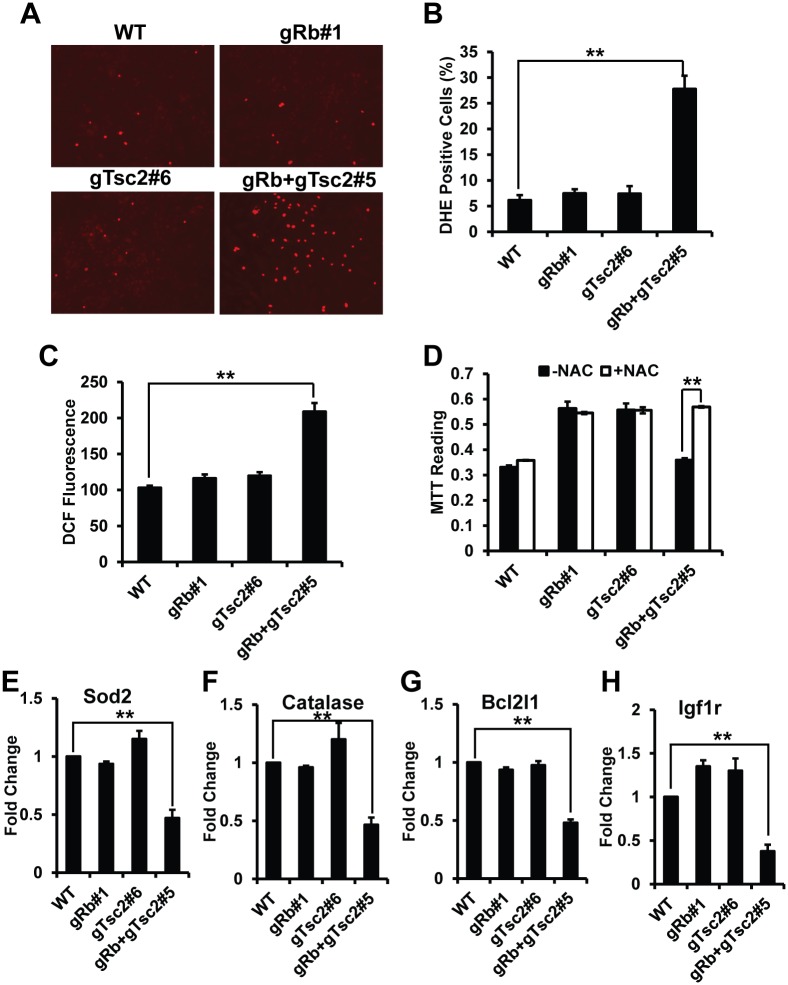
Enhanced Rb/E2F and TSC/mTOR pathways induce oxidative stress in VSMCs. (A) Cell stained with Dihydroergotamine (DHE) showing increased DHE fluorescence in Rb and TSC2 double knockout cells; (B) Quantification of the percentage of DHE positive cells in (A); (C) Cell stained with H2DCFDA showing increased DCF fluorescence in cells with Rb and TSC2 double knockout analyzed by flow cytometry; (D) Cell proliferation measured by MTT assay showing increased cell growth in cells with Rb and TSC2 double knockout after treated with N-acetyl cysteine (NAC, 5mM) in comparison to control cells; (E-H) mRNA expression of Sod2 (E), Catalase (F), Bcl2l1 (G) and insulin-like growth factor 1 receptor (Igf1r) (H) relative to the level of GAPDH in cells with Rb and TSC2 double knockout. **<0.01.

Previous studies showed that the synergistic cell death induced by enhanced E2F and mTOR activities is due to increased cellular stress, especially oxidative stress [10]. So we hypothesized that the synergistic inhibition of VSMC proliferation by enhanced E2F and mTOR activities might be related to higher level of oxidative stress. To test it, we first used Dihydroergotamine (DHE), a dye that detects superoxide, to determine whether knockout Rb and TSC2 induces oxidative stress in PDGF-induced VSMCs. We found that cells with single knockout of Rb or TSC2 showed similar percentage of DHE positive cells as control cells. However, cells with double knockout of Rb and TSC2 have significantly enhanced DHE fluorescence than control cells (Fig 4A). The percentage of DHE positive cells is significantly higher than control cells (Fig 4B). We next use another fluorescent dyes H2DCFDA to monitor the ROS level in cells. H2DCFDA was used as an indicator for ROS in cells. We found cells with double knockout of Rb and TSC2 have significantly enhanced DCF fluorescence than control cells (Fig 4C). Therefore, enhanced E2F and mTOR activities induce oxidative stress in VSMCs.

The above observation suggests that inhibition of VSMCs proliferation by enhanced E2F and mTOR activities is due to synergistic increased oxidative stress. To test this idea, the antioxidant N-acetyl cysteine was used to reduce oxidative stress. Addition of N-acetyl cysteine significantly rescued the proliferation defect in cells with Rb and TSC2 double knockout (Fig 4D). The observation strongly supports that enhanced E2F and mTOR activities synergistically induced oxidative stress, and further inhibit the PDGF-induced proliferation in VSMCs.

To study how enhanced E2F and mTOR activities synergistically induced oxidative stress and inhibited proliferation in VSMCs, we first measured the gene expression of ROS scavenger enzymes in cells. SOD2 and catalase are two well-known ROS scavenger enzymes. We found that the expressions of Sod2 and catalase were significantly inhibited in cells with Rb and TSC2 double knockout (Fig 4E and 4F). In cancer cells, the synergistic effect induced by enhanced E2F and mTOR activities changed the gene expression of some components in cell proliferation/survival signaling, such as Bcl2l1 and insulin-like growth factor 1 receptor (Igf1r). So we next tested the gene expression of Bcl2l1 and Igf1r in cells. The data showed that the expressions of Bcl2l1 and Igf1r were significantly inhibited in cells with Rb and TSC2 double knockout (Fig 4G and 4H). These data suggest that enhanced E2F and mTOR activities synergistically regulate genes expression to induce ROS stress and to inhibit proliferation in VSMCs.

## Discussion

Although evidences from previous studies suggest the roles of Rb/E2F and TSC/mTOR pathways in the proliferation of VSMCs, its precise contribution in PDGF-induced proliferation has been less studied. In this study, we demonstrated the roles of Rb/E2F and TSC/mTOR pathways in PDGF-induced proliferation in VSMCs. Consistent with important roles in the PDGF-induced proliferation, both E2F and mTOR activities were induced right after treatment of PDGF. And enhancing either E2F or mTOR activity increases cell proliferation and DNA synthesis. The enhanced proliferation may be due to the increased level of cell cycle proteins. It is well known that Rb/E2F pathway is the major regulator of cell cycles. And cell cycle proteins were also reported to be regulated through PI3-k and mTOR in VSMCs. Studies found that p70 (S6) kinase, a target of PI3-kinase and the mTOR, was induced in quiescent coronary artery smooth muscle cells after stimulation of growth factors. The events were associated with increased protein level of cell cycle proteins like cyclin A2, cyclin-dependent kinase 1 and others [13]. Many studies linked cell cycle proteins to PDGF-induced proliferation in VSMCs. Expression of dominant-negative mutants of ERK or JNK attenuated PDGF-BB-induced VSMCs proliferation through modulating cell cycle proteins [14].

Remarkably, we revealed that increasing both E2F and mTOR activity leads to synergistic inhibition of PDGF-induced proliferation in VSMCs. We further identified that the cause of the synergistic inhibition is the enhanced oxidative stress. In cancer cells, knockdown of Rb and TSC2 induced synergistic cell death. The cell death is due to enhanced cell stress [10]. Different to cancer cells, no significant cell death effect was found in VSMCs after knockdown of Rb and TSC2. It seems the synergistic cell effect is less strong in VSMCs. It might due to different tolerance to cellular stresses in the cells. Different to VSMCs, cancer cells generally exhibit increased cellular stresses and are more dependent on stress support pathways for survival. This makes cancer cells more susceptible to either stress sensitization or stress overload. However, considering the important normal physiological functions of VSMCs, the synergistic growth inhibition effect might be more meaning for curing diseases caused by the unregulated proliferation of VSMCs, such as atherosclerosis, in-stent restenosis, transplant vasculopathy, and vein bypass graft failure.

Although ROS have been shown to function as important pathway molecules in promoting proliferation in VSMCs, some conflicting results have been reported [15,16,17]. Previous studies showed ROS induced apoptosis in a concentration-dependent manner in VSMCs. H_2_O_2_ increased DNA synthesis, but was followed by cell death [18]. VSMCs exposed to glucose oxidase or diethylmaleate induced apoptosis via the formation of hydroxyl radicals [19]. So it is not surprising to find that enhanced ROS level leads to synergistic growth inhibition instead of promoting growth in VSMCs. The important factor of final effect is the magnitude of alteration in the redox state. Although a certain level of oxidant stress appears to be growth-promoting, more severe stress might lead to growth inhibition or cell death. However, the mechanism by which cellular stresses induced growth inhibition in VSMCs is complex and is not entirely clear. In our study, we found that enhanced E2F and mTOR activities synergistically regulate genes expression of ROS scavenger enzymes and components in cell survival/proliferation signaling. In cancer cells, studies found that the oxidative stress might result from other stresses, such metabolic stress and ER stress, which are induced by knockdown of Rb and TSC. Further studies are required to reveal the details.

In conclusion, our studies show that Rb/E2F and TSC/mTOR pathways regulate PDGF-induced proliferation in VSMCs. Enhancing both E2F and mTOR activity leads to synergistic inhibition in cell proliferation. The synergistic inhibition effect could provide a potential way to against vascular diseases caused by the deregulation of VSMCs proliferation.
